# Tobacco-Derived and Tobacco-Free Nicotine cause differential inflammatory cell influx and MMP9 in mouse lung

**DOI:** 10.21203/rs.3.rs-3650978/v1

**Published:** 2023-11-28

**Authors:** Thomas Lamb, Gagandeep Kaur, Irfan Rahman

**Affiliations:** University of Rochester Medical Center; University of Rochester Medical Center; University of Rochester Medical Center

**Keywords:** E-cigarettes, Flavors, synthetic nicotine, natural nicotine, lungs

## Abstract

Electronic nicotine delivery systems (ENDS) or electronic cigarettes (e-cigarettes) have propylene glycol (PG) and vegetable glycerin (VG) as humectants, flavoring chemicals, and nicotine. Nicotine naturally occurs in two isomers R- and S-nicotine, with both tobacco-derived nicotine (TDN) composed of S-nicotine and synthetic nicotine (TFN) composed of a racemic mixture of R- and S-nicotine. Currently there is limited knowledge of the potential differences in the toxicity of TFN vs TDN. We hypothesized that exposure of TFN salts to C57BL/6J mice will result in a differential response in inflammation and lung protease and antiprotease imbalance compared to TDN salts exposed mice. We studied the toxicological impact of these isomers by exposing mice to air, PG/VG, PG/VG with TFN salts, or PG/VG with TDN salts by nose-only exposure and measured the cytokine levels in BALF and lung homogenate along with MMP protein abundance in the lungs of exposed mice. Exposure to the humectants, PG/VG, used in e-cigarettes alone was able to increase cytokine levels-IL-6, KC, and MCP-1 in BALF and KC levels in lung homogenate. Further, it showed differential responses on exposure to PG/VG with TDN salts and PG/VG with TFN salts since PG/VG with TDN salts did not alter the cytokine levels in lung homogenate while PG/VG with TFN salts resulted in an increase in KC levels. PG/VG with TDN salts increased the levels of MMP9 protein abundance in female exposed mice, while PG/VG with TFN salts did not alter MMP9 levels in female mice. The metabolism of nicotine or the clearance of cotinine from TFN may differ from the metabolism of nicotine or the clearance of cotinine from TDN. Thus exposure of humectants alone to induce an inflammatory response while PG/VG with TFN salts and PG/VG with TDN salts may differentially alter inflammatory responses and lung proteases in acute exposures. These data suggest the harmful effects of synthetic/natural nicotine and PG/VG and potential toxicological risk for users.

## Introduction

Ever since the February 2020 ban by the US Food and Drug Administration (FDA) on the flavored cartridge-based electronic cigarettes (e-cigs), the national sales of disposable e-cigarette device have increased drastically [1]. Shortly afterwards, flavored products claiming to contain ‘tobacco free nicotine’ (TFN) entered the markets. These products are being advertised as; ‘cleaner’, ‘purer’, ‘tastier’ and ‘having higher quality’, as compared to traditionally available ‘tobacco-derived nicotine’ (TDN) products. While these strategies have benefitted the sale of flavored e-cig products, they have also created confusion as many users believe them to contain ‘no tobacco’. Prior to April 2022, there was a regulatory gap that meant the FDA could not regulate TFN since it is not made or derived from tobacco which allowed tobacco companies to keep products on the market without needing to go through the pre-market approval process [1, 2]. In April 2022, this regulatory gap was closed after the new legislation allowed the FDA to regulate TFN products [1]. Yet, there is little to no knowledge with regards to the toxic effects of the use of TFN-containing products.

Nicotine is a naturally occurring alkaloid extracted from tobacco leaves that exists in two isomers: (R)-(+)-nicotine and (S)-(−)-nicotine [3, 4]. Traditionally tobacco products have utilized nicotine extracted from tobacco leaves which contains predominantly S-nicotine [5, 6]. TFN, on the other hand, is a chemically synthesized nicotine containing a racemic (50:50) mixture of R- and S-forms.[5]

Besides these two isomers, nicotine can also be found in two forms- a protonated and a free base form [8]. The protonated form of nicotine is formed from the addition of acid to freebase nicotine to form nicotine salts. The two most commonly used acids for this purpose being lactic and benzoic acid [9]. Nicotine salts in e-liquids are found to have a lower pH level than freebase nicotine even with higher levels of nicotine present [10]. This reduction in pH is believed to reduce the throat irritation and harshness due to high levels of nicotine[11]. In clinical studies, protonated nicotine has been found to result in a higher nicotine absorption than free-base nicotine, although, contrary evidence exists though *in vivo* and *in vitro* studies, thus warranting further investigation [8, 12].

Nicotine is known to have adverse effects on the respiratory system [13]. Previous work within our lab using freebase nicotine, has shown that acute exposure (3 days; 2 hr/day) of C57BL/6J mice to PG with nicotine resulted in an increase in pro-inflammatory cytokines and MMP2 levels in exposed mice as compared to the air controls [14]. Furthermore, sub-chronic (3 weeks; 3 h/d; 5 d/wk) exposures of C57BL/6J mice to PG/VG with freebase nicotine has also demonstrated sex-specific alterations in lipogenic and myogenic gene expression and levels of matrix metalloproteinase 9 (MMP9) [15]. Our lab has also previously conducted research on e-cigarettes with nicotine salts, and has found pods containing nicotine salts result in an increase in pro-inflammatory cytokines, oxidative stress, epithelial barrier dysfunction and DNA damage in lung epithelial cells [16]. These results provide evidence for the adverse toxicological effects of both free-form and protonated form of nicotine. However, there is limited knowledge about the pharmacological effects of R-nicotine. Current data has indicated that R-nicotine is a less potent agonist of nicotinic acetylcholine receptors andcan bind and inhibit acetylcholinesterase. Further, R-nicotine and R-cotinine has been found to have a faster clearance than S-nicotine or S-cotinine [5, 17, 18].

We thus hypothesized that exposure of C57BL/6J mice to TFN salts will result in a differential response in inflammation and lung protease/antiprotease imbalance compared to TDN salts exposed mice. To test this hypothesis, we exposed mice to air, PG/VG, PG/VG with TFN salts, or PG/VG with TDN salts, and measured the cytokine levels in BALF and lung homogenate along with MMP protein abundance in the lungs of exposed mice.

## Materials and Methods

### Ethics Statement

Experiments were conducted using the standards established by the United States Animal Welfare Act. Animal experimental protocols conducted at the University of Rochester were approved by the University Committee on Animal Resources. All laboratory studies were approved by the Institutional Biosafety Committee of the University of Rochester Medical Center.

### Mouse Exposures

An equal number of male (12) and female (12) C57BL/6J mice at five weeks old were ordered from Jackson Laboratory. Mice were housed for one week at the University of Rochester Vivarium prior to being moved to the inhalation suite to begin nose-only tower training. In order to acclimatize the mice to the mesh restraints of the nose-only tower, one week prior to beginning the e-cig exposure mice were placed in the mesh restraints and held in the tower. Mice were trained for five days following the methodology described in Lamb, *et al*. [19]. The first day mice were held in the restraints for fifteen minutes, the second day mice were held in the restraints for thirty minutes, the third day mice were held in the restraints for forty-five minutes, and the final two days mice were held in the restraints for one hour.

### E-cigarette Device and E-liquid

A Joyetech eVic-VTC mini and cubis pro atomizer (SCIREQ, Montreal, Canada), with a BF SS316 1.0 Ω coil (Joyetech, Shenzhen, China) and the Scireq nose-only tower (SCIREQ, Montreal, Canada) were utilized for all e-cigarette exposures. PG and VG from “EC Blend” were purchased through local vendors/online vendors. A 1:1 mixture of PG/VG was used for PG/VG exposures and a 1:1 mixture of PG/VG mixed with a 1:1 mixture of lactic acid with R-nicotine ((±)-Nicotine, Sigma-Aldrich, Cat# N0267) or S-nicotine ((−)-Nicotine, Sigma-Aldrich, Cat#N3876) at a concentration of 50 mg/mL was used for PG/VG with TFN salts and PG/VG with TDN salts exposure, respectively. Nicotine e-liquids utilized for exposures were analyzed by proton nuclear magnetic resonance (^1^H NMR) to confirm nicotine concentration and R/S ratio. In brief ^1^H NMR following the same basic methodology as described in Lamb, *et al*. was used to determine nicotine concentrations for PG/VG with TFN salts at 52.09 mg/mL and for PG/VG with TDN salts at 48.35 mg/mL [19]. The ratio of R-nicotine to S-nicotine was determined by ^1^H NMR following a similar methodology as described in Duell, *et al*. and determined the R/S ratio of nicotine in PG/VG with TFN salts to be 56/44 and in PG/VG with TDN salts to be 0/100 [20].

### E-cigarette Exposure

Nose-only e-cigarette exposure was conducted utilizing the Scireq InExpose system with the Scireq flexiware software controlling the Joyetech eVic-VTC mini device. Mice were exposed to a puffing profile of two puffs per minute with a puff volume of 51 mL, puff duration of three seconds, and an inter puff interval of twenty seven seconds with a 2 L/min bias flow between puffs [21]. Mice were split into four groups-(i) air, (ii) PG/VG, (iii) PG/VG with 50 mg/mL TDN salts (labelled as TDN), and (iv) PG/VG with 50 mg/mL TFN salts (labelled as TFN), of equal number of male (3) and female (3) mice. Mice were exposed to the described puffing profile for one hour per day (120 puffs) for a total of five days, with air mice being exposed to room air [22]. Temperature, humidity, and CO levels were measured at the starting, mid-, and end point of the exposure utilizing Q-Trak Indoor Air Quality Monitor (TSI, SKU#7575). Total particulate matter (TPM) measurements were taken at the exhaust tubing at the half-way point of the exposure and at the inlet tubing connected to the top of the nose-only tower immediately after the end point of the exposure. TPM was measured by weighing a glass fiber filter pad (Pall Corporation, P/N#61630) before and after collecting aerosol over the course of five minutes. Cotinine levels in the blood serum of exposed mice were measured using an ELISA based assay (Calbiotech, Cat#C0096D) following manufacturer’s protocol.

### Mouse Sacrifice

Mice were sacrificed two hours after the final e-cigarette exposure and were anesthetized with a mixture of ketamine and xylazine. Blood was drawn from the inferior vena cava, and allowed to sit for roughly thirty minutes before being centrifuged at 2000 rpm for fifteen minutes. After being centrifuged, the serum was collected and stored at −80°C. Manual lung perfusion was performed by taking 3 mL of 1x PBS in a 3 mL syringe and slowly injecting the 1x PBS into the heart of the mouse until the lung lobes turned white. Mice were lavaged via catherization three separate times with 0.6 ml of 0.05% FBS in 0.9% NaCl. The combined lavage fluids were centrifuged at 3000 rpm for 10 minutes at 4°C. The supernatant was recovered and stored at −80°C until further experimentation, while the cell pellet was re-suspended in 1 ml of 1x PBS for determination of immune cell population. Mouse lung lobes were harvested from exposed mice and washed with 1x PBS, one lobe was left in 1 mL 1x PBS while the rest were blotted dry using a filter pad, and then flash frozen by dry ice before being stored at −80°C.

### Lung Digest, Cell Count, and Flow Cytometry

Lung lobes to be used for lung digestion were minced finely, and placed into a 50 mL conical tube with a liberase enzymatic cocktail (0.5 mL of 5 mg/mL liberase with 2 mL DMEM and 3 μL of 100 mg/mL DNAse I). Tissue samples were dissociated using gentleMACS Dissociator (Miltenyi, Biotec, Gaithersburg, MD), placed on a rocker and incubated at 37°C for thirty minutes. After incubation, cell suspensions were strained through a 70 micron cell strainer into a new 50 mL conical tube and then remaining cells were collected by adding 5 mL DMEM (10% FBS) through the cell strainer. Cells were spun at 300 g for five minutes at 4°C, afterwards, the supernatant was removed and 1 mL of RBC lysis buffer was added to the cell pellet and incubated on ice for one minute. After incubation 5 mL of DMEM (10% FBS) was added to the cell suspension and then centrifuged at 300 g for five minutes at 4°C. Supernatant was removed and the cell pellet was re-suspended in 2 mL DMEM (10% FBS). Total cell counts for BALF and lung digest were measured by staining cells with AO/PI and counted using the Nexcelom Cellometer Auto 2000 cell viability counter. Differential cell counts were determined by flow cytometry using the BD LSRFortessa cell analyzer. Cells from both lung digest and BALF were stained with CD16/32 (Cat#70–0161-u500, Tonbo Biosciences, 1:10 dilution) to block nonspecific binding and then stained with a master mix of Siglec F (Cat#740280, BD Biosciences, 1:200 dilution), CD11b (Cat #101243, Biolegend, 1:200 dilution), Ly6G (Cat# 562700, BD Biosciences, 1:200 dilution), CD45 (Cat#103126, Biolegend, 1:200 dilution), and CD11c (Cat #117318, Biolegend, 1:200 dilution), with 7AAD (Cat#00–6993-50, eBiosciences, 1:10 dilution) being added just prior to analysis by flow cytometry with cells used for compensation.

### Protein Extraction

Roughly 20–30 mg of flash frozen lung tissue was added to 350 μL of RIPA buffer containing protease inhibitor (Cat#87785, Thermo Fisher Scientific) and EDTA (Cat#R1021, Thermo Fisher Scientific) and mechanically homogenized while on ice. After homogenization, samples remained on ice for forty-five minutes and then spun at 14000 rpm for thirty minutes at 4°C. The supernatant was collected and 50 μL aliquots were stored at −80°C. Total protein concentration for each sample was determined using the Pierce BCA Protein Assay kit (Cat#23225, Thermo Fisher Scientific) with BSA being utilized as the protein standard.

### Pro-Inflammatory Cytokines/Chemokines Levels

Pro-inflammatory cytokine/chemokine KC (R&D DuoSet DY453), IL-6 (R&D Duoset DY406), and MCP-1 (R&D DuoSet DY479) levels were measured using ELISA following manufacturer’s protocol in BALF and lung homogenate. A dilution of 1:10 was utilized for lung homogenate samples and no dilution was utilized for BALF samples. Lung homogenate was normalized to total protein amount of each sample.

### Immunoblot Assay

An equal concentration of protein (10 μg) from each lung homogenate samples were loaded per well of a 26 well 4–15% Criterion Precast Gel (Cat#5671085, BioRad) with 10 μl of Precision Plus Protein^™^ Kaleidoscope^™^ Prestained Protein Standards (Cat# #1610375, BioRad) added to the first well. Protein were separated based on size using gel electrophoresis before being transferred to a nitrocellulose membrane. Membranes were blocked for one hour with 5% BSA in 1x TBST or with 5% non-fat milk in 1x TBST at room temperature. Membranes were probed with primary antibodies, using the following antibodies diluted in 1% BSA in 1x TBST: TIMP-1 (Cat#ab179580, Abcam, 1:1000), and the following antibodies diluted in 5% non-fat milk in 1x TBST: MMP2 (Cat# ab92536, Abcam, 1:1000), MMP9 (Cat# ab38898, Abcam, 1:1000), and MMP12 (Cat# NBP2–67344, Novus Biological, 1:1000) and were left rocking overnight at 4°C. After membranes were incubated overnight, membranes were washed with 1x TBST and then incubated with a goat anti-rabbit secondary antibody (Cat#1706515, BioRad, 1:10000) in 5% BSA in 1x TBST or 5% non-fat milk in 1x TBST. After incubation with secondary antibody, membranes were washed with 1x TBST and then signals were measured using West Femto Maximum Sensitivity Substrate (Cat#34096, Thermo Fisher) following the manufacturer’s protocol. Images of membranes were collected utilizing the Bio-Rad ChemiDoc MP Imaging system (Bio-Rad Laboratories). After imaging, membranes were stripped utilizing restore western stripping buffer (Cat#21063, Thermo Fisher ) and re-probed with antibodies for the other proteins and finally for GAPDH (Cat# 2118S, Cell Signaling, 1:1000). Band intensity was determined using densitometry analysis using image lab software and normalized to the levels of GAPDH. Fold change in protein abundance were relative to the protein abundance of air exposed mice.

### Gelatin Gel Zymography

Total MMP activity levels were measured by following the gelatin zymography protocol from Abcam with slight modifications. The night prior to the start of the assay, the gelatin gel was prepared with a 15-well comb utilizing the Mini-PROTEAN Tetra Cell Casting Module (Cat#1658022 BioRad). After the gels had solidified, the gels were left at 4°C in 1x running buffer. An equal concentration of protein (50 μg) from each lung homogenate samples were loaded per well of the gelatin gel, with 10 μl of Precision Kaleidoscope Prestained Protein Standards (Cat# #1610375, BioRad) added to the first well. Proteins were separated based on size through the stacking gel and separating gel. Once the protein are separated, the gels were washed with washing buffer two times for thirty minutes, rinsed twice with incubation buffer for 10 minutes per rinse while rocking, and then fresh incubation buffer was added to cover the gels which were then incubated for twenty-four hours at 37°C. After incubations, the gels were stained with staining solution for one hour with rocking at room temperature. After staining, the gels were rinsed with ddH_2_O and then destaining solution was added and incubated until bands could be visualized. Images of membranes were collected utilizing the Bio-Rad ChemiDoc MP Imaging system (Bio-Rad Laboratories). After imaging, band intensity was determined using densitometry analysis using image lab software and fold change in activity levels were relative to the activity levels of air exposed mice.

### Statistical Analysis

Analysis was performed using GraphPad Prisma utilizing One-Way ANOVA with Tukey’s multiple comparisons test with data shown as mean ± SEM.

## Results

### PG/VG with synthetic nicotine salts exposure alters inflammatory cells infiltration in exposed mice.

In order to determine the effects of TFN and TDN salts on inflammatory cell influx in vivo, C57BL/6J mice were exposed to nose-only exposure to aerosols from PG/VG, TFN and TDN salts as described earlier. Average TPM measurements at the inlet for for all the exposures was comparable with the values being: 2755.33 mg/m^3^, 3300.67 mg/m^3^ for TFN, and 3367.00 mg/m^3^ for PG/VG, TFN and TDN exposures respectively. We further performed Cotinine assay to ensure exposure to tobacco in our samples and found the levels of cotinine in the blood serum to be significantly varied between TFN (53.69 ± 9.63 ng/mL) and TDN (104.41 ± 28.09 ng/mL) exposed mice (**Supplementary Fig. 1A**).

**Figure 1:**
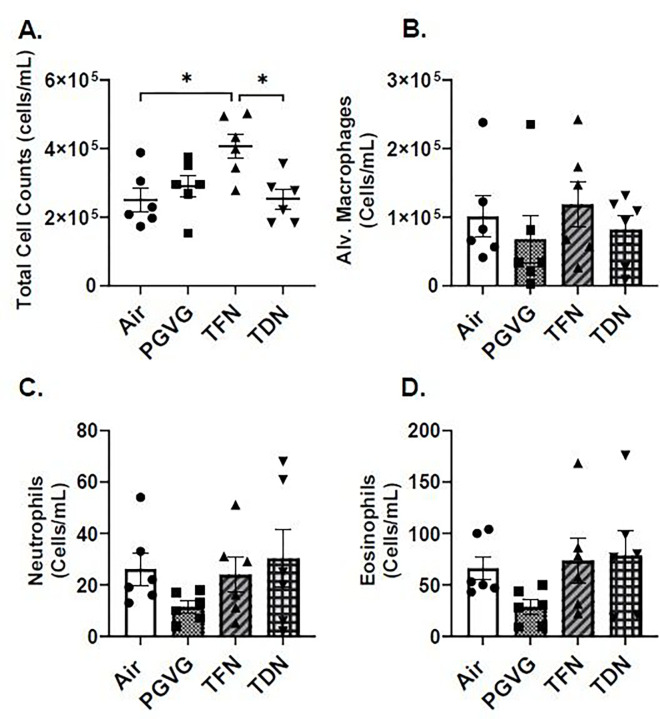
Differential effects of synthetic and tobacco-derived nicotine salts on infiltrating inflammatory cells in BALF. Mice were exposed to air, PG/VG, PG/VG with TFN salts (TFN), and PG/VG with TDN salts (TDN) for five days for one hour per day. Mice were sacrificed two hours after final exposure. (A) Total cell counts were measured in BALF by staining cells with AO/PI and counted with a cellometer. Flow cytometry was performed to determine the number of(B) Alveolar Macrophages (CD45^+^Siglec F^+^CD11b^−^), (C) Neutrophils (CD45^+^Siglec F^−^CD11b^+^Ly6G^+^), (D) Eosinophils (CD45^+^CD11b^+^Ly6G^−^CD11c^−^Siglec F^+^) in BALF of control and experimental groups. Data represented as mean ± SEM and analyzed using one-way ANOVA with Tukey’s multiple comparison with * p < 0.05, N = 6.

Lung inflammation following acute exposures to TFN and TDN salts was determined using flow cytometry. Interestingly, we observed a significant increase in the total cell counts in BALF from TFN exposed mice as compared to the mouse exposed to TDN salts pointing towards elicitation of varied immune responses in C57Bl/6J mice on exposure to TFN salts versus TDN (Fig. 1A). Mice exposed to PG/VG, TFN salts, and TDN salts did not alter the differential cell counts of alveolar macrophage, eosinophils or neutrophils in the BALF compared to air exposed mice (Fig. 1B–D). We did not observe any change in the differential cell counts of alveolar macrophages, neutrophils and eosinophils in the lung homogenates from TFN and TDN exposed mouse lungs as compared to air controls. It is pertinent to mention, though, that the neutrophilic responses in the lung tissues of TFN exposed mice (73487 ± 55121.28) was higher than those exposed to TDN (28523 ± 5202.428) (Supplementary Fig. 1B-D).

### PG/VG exposure alters inflammatory cytokines in BALF

**Figure 2:**
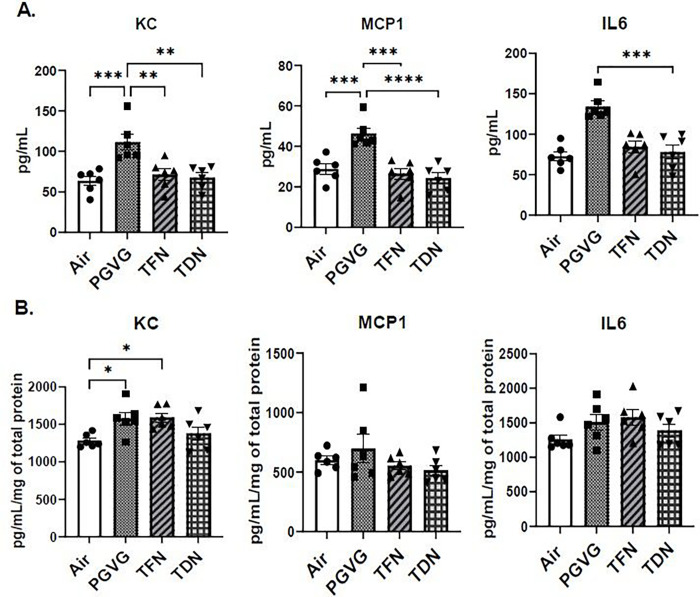
Differential effects of synthetic and tobacco-derived nicotine salts on pro-inflammatory cytokine production. Mice were exposed to air, PG/VG, PG/VG with TFN salts (TFN), and PG/VG with TDN salts (TDN) for five days for one hour per day. Mice were sacrificed two hours after final exposure. Pro-inflammatory cytokines, IL-6, KC, and MCP-1 were measured in (A) BALF and (B) lung homogenate. Data represented as mean ± SEM and analyzed using one-way ANOVA with Tukey’s multiple comparison with * p < 0.05, ** p < 0.01, *** p < 0.001, and **** p < 0.0001, N = 6.

In order to determine the potential of TFN and TDN salts exposure to induce an inflammatory response, pro-inflammatory cytokines were measured in BALF and lung homogenate. Mice exposed to PG/VG resulted in a significant increase in IL-6, KC, and MCP-1 levels in BALF compared to air, TFN, and TDN salts exposed mice (Fig. 2A). Contrarily, mice exposed to PG/VG, TFN, or TDN salts did not significantly alter IL-6 and MCP-1 levels in lung homogenate compared to air exposed mice (Fig. 2B). Mice exposed to PG/VG and TFN salts significantly increased KC levels compared to air exposed mice (Fig. 2B).

### PG/VG with tobacco-derived nicotine salts alters MMP9 protein abundance

**Figure 3:**
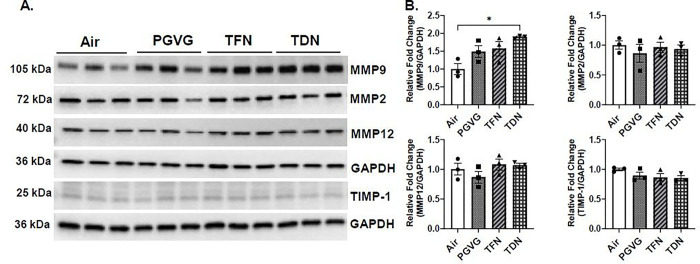
Differential effects of synthetic and tobacco-derived nicotine salts on MMPs protein abundance in female mice. Mice were exposed to air, PG/VG, PG/VG with TFN salts (TFN), and PG/VG with TDN salts (TDN) for five days for one hour per day. Mice were sacrificed two hours after final exposure. The protein abundance of MMP2, MMP9, MMP12, and TIMP-1 was measured in lung homogenate with GAPDH used as a loading control by western blot. (A) Representative images for MMP2, MMP9, MMP12, TIMP-1, and GAPDH for exposed female mice. (B) Band intensity was measured by densitometry with relative fold change being measured compared to air exposed female mice. Data represented as mean ± SEM and analyzed using one-way ANOVA with Tukey’s multiple comparison with * p < 0.05, N = 3. Images of full blots can be found in Supplementary Figure 2 – 7.

**Figure 4:**
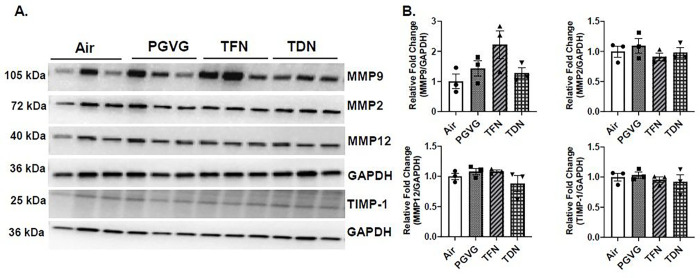
Differential effects of synthetic and tobacco-derived nicotine salts on MMPs protein abundance in male mice. Mice were exposed to air, PG/VG, PG/VG with TFN salts (TFN), and PG/VG with TDN salts (TDN) for five days for one hour per day. Mice were sacrificed two hours after final exposure. The protein abundance of MMP2, MMP9, MMP12, and TIMP-1 was measured in lung homogenate with GAPDH used as a loading control by western blot. (A) Representative images for MMP2, MMP9, MMP12, TIMP-1, and GAPDH for exposed male mice. (B) Band intensity was measured by densitometry with relative fold change being measured compared to air exposed male mice. Data represented as mean ± SEM and analyzed using one-way ANOVA with Tukey’s multiple comparison with N = 3. Images of full blots can be found in Supplementary Figure2 – 7.

In order to determine the effect of PG/VG with TFN and PG/VG with TDN salts to alter lung protease levels, protein abundance of MMPs was measured. In PG/VG exposure, both male and female mice had no change in MMP2, MMP9, MMP12, or TIMP-1 protein abundance compared to air exposed mice (Figs. 3, 4). In TFN salt exposed mice, both female and male mice had no change in MMP2, MMP9, MMP12, or TIMP-1 protein abundance compared to air exposed mice (Figs. 3, 4). In TDN salt exposed mice, both male and female mice had no change in MMP2, MMP12, or TIMP-1 protein abundance compared to control mice. In female mice exposed to PG/VG with TDN salts a significant increase in MMP9 protein abundance was observed compared to air exposed mice while no change in the MMP9 protein abundance was observed in male mice exposed to TDN salts compared to air controls (Fig. 3, 4).

### PG/VG with tobacco-derived nicotine salts alters MMP9 and MMP2 activity levels

**Figure 5:**
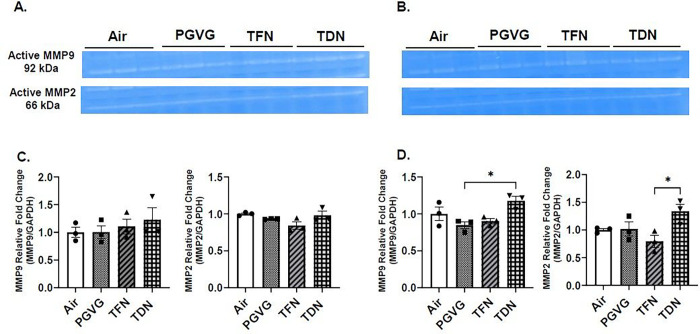
Differential effects of synthetic and tobacco-derived nicotine salts on MMP9 and MMP2 activity level. Mice were exposed to air, PG/VG, PG/VG with TFN salts (TFN), and PG/VG with TDN salts (TDN) for five days for one hour per day. Mice were sacrificed two hours after final exposure. The activity of MMP2 and MMP9 was measured in lung homogenate by gelatin gel zymography. Representative images for MMP2 and MMP9 for exposed (A) female and (B) male mice. Band intensity was measured by densitometry with relative fold change being measured compared to air exposed (C) female and (D) male mice. Data represented as mean ± SEM and analyzed using one-way ANOVA with Tukey’s multiple comparison with * p < 0.05, N = 3. Images of full gelatin gels can be found in Supplementary Figure 8.

In order to determine the effects of PG/VG with TFN and PG/VG with TDN salts to alter lung protease activity, total activity levels of MMP2 and MMP9 were determined using gel zymography. In PG/VG exposure, both male and female mice did not result in a significant change in MMP2 or MMP9 activity levels compared to air exposed mice (Fig. 5). In TFN salt exposed mice, both male and female mice did not result in a significant change in MMP2 or MMP9 activity levels compared to air exposed mice (Fig. 5). In TDN salt exposed mice, MMP9 activity levels were significantly increased in female mice, whereas no change was observed in the males as compared to mice exposed to PG/VG only (Fig. 5). Furthermore, TDN salts exposure resulted in a significant increase in MMP2 activity levels compared to TFN salts exposed female mice; while no change was observed for male mice (Fig. 5).

## Discussion

Although TFN has only recently begun to be used in tobacco products, TFN has been around for many years. However, unlike TDN, the health effects of TFN salts are relatively unknown [5, 7, 17, 18]. This study attempted to understand the potential toxicological differences between tobacco-derived nicotine (TDN) and synthetic nicotine (TFN). PG/VG with TFN salts and PG/VG with TDN salts were found to be at concentrations of roughly 50 mg/mL in e-liquids. Despite the similar exposures between the two exposure groups, there is a significantly increased level of serum cotinine in PG/VG with TDN salts exposed mice. This difference in serum cotinine levels indicates the possibility that TFN may be metabolized in mice differently than TDN. While there is limited knowledge about the metabolism and clearance of R- and S-cotinine in humans, a 1988 study studied the disposition kinetics of nicotine and cotinine enantiomers in rabbits and beagle dogs. This study showed that while the clearance of R- and S-nicotine differed in beagle dogs; the clearance of R-cotinine in rabbits was twice that of S-cotinine [18]. This substantiates our claim that biotransformation of the two enantiomers could be different for the TFN versus TDN and requires further research. Besides the differences in cotinine levels between TFN salts and TDN salts, serum cotinine levels in the air and PG/VG only exposure groups indicates that alteration in inflammatory cytokines and protease levels are due to exposure to the humectants alone.

Our results found that PG/VG with TFN salts increased total cell counts in the BALF but did not alter macrophage, neutrophil or eosinophil cell counts in BALF. While we looked into the myeloid cell population of immune cells in this study, there is a possibility that the exposures to TFN salts results in an increase in the lymphocytic (B-cell and T-cell) populations in these mice that could explain the significant increase in the total cell counts in mice exposed to TFN salts compared to controls. Future work in this area might be able to shed more light into this mechanism. Though not significant, the exposure to TFN salts resulted in a 1.6 fold increase in the neutrophil cell count in the lung homogenate as compared to the TDN salt exposed mouse lungs. This proves that the immunological responses on exposure to both TFN and TDN salts are distinct. Although our study found no change in eosinophil levels in either nicotine exposures, we observed a decrease in eosinophil count in lung digest of PG/VG exposed mice. This is contrary to previous observations. Chapman, *et al*., found that exposure of Balb/c mice to flavored e-liquids with nicotine resulted in a significant decrease in the eosinophil cell counts after treatment with house dust mites [26]. While Ahmad, *et al*., found that exposure of nicotine aerosol to male Sprague-Dawley rats led to elevated levels of eosinophil counts in the blood of exposed rats [27]. Although these exposures found contrary results, this may be due to different puffing topography, a higher temperature set for the heating of e-liquids, or the use of a nicotine solution not within PG or VG, indicating that the alterations in exposure methodologies can result in differential responses in rodents. Furthermore, it is also important to note that each of these studies used different mouse strains which may not show comparable results as each strain has a distinct innate immune response [28].

Our results found that exposure to TDN salts and TFN salts had a significant decrease in IL-6, KC, and MCP-1 levels compared to exposure PG/VG alone exposed mice. Similar to these results, other investigations that utilized male Balb/c mice treated with lipopolysaccharide (LPS) by intratracheal administration and treated with nicotine by intraperitoneal administration, resulted in a decrease in IL-1, IL-6, and TNF-α cytokine levels in BALF compared to LPS treated alone mice [29]. Glynos, *et al*., exposed male C57BL/6J mice to PV/VG with nicotine similarly resulted in no alteration in lung homogenate cytokine levels of IL-1β, TNF-α, and IL-6 at an acute and sub-chronic exposure time points [30]. Although our study and previous work have shown anti-inflammatory effects of nicotine, contradictory evidences also exist. Ahmad, *et al*., found that male Sprague-Dawley rats exposed to nicotine aerosols results in an increase in the mRNA expression of IL-1α and CXCL1 along with increases in IL-1α protein levels [27].

Garcia-Arcos, *et al*. demonstrated an increase in the mRNA expression of IL-1β, MCP-1, and IL-6 in A/J mice exposed to PG/VG with nicotine as compared to PG/VG only [31]. These variations from our results and between previous studies could be a result of variations in the puffing topography and exposure duration. Further, these studies utilize free-base TDN while our study used both TDN and TFN salts for exposure which could explain the obtained results. Nevertheless, our results indicate that nicotine salts may have altered effects compared to free-base nicotine exposures.

PG/VG alone exposure resulted in an increase in cytokine levels of KC, IL-6, and MCP-1 in BALF. Previous studies have also show in a significant increase in the IL6 and IL8 production in lung epithelial cells (16-HBE), exposed separately to propylene glycol or vegetable glycerin only [32], thus indicating that base humectants alone may pose a risk to e-cigarette users.

Our results showed that exposure to PG/VG and PG/VG with TFN salts did not alter the protein abundance of MMPs or TIMP-1 levels while PG/VG with TDN salts did increase MMP9 protein abundance in female mice. A previous study has reported an increase in the mRNA expression of MMP9 and MMP12, along with other lung proteases in Cathepsin K and Cathepsin L1 in PG/VG with nicotine exposed study A/J mice as compare to only PG/VG exposures [31]. Similarly another study, demonstrated an increase in MMP9 and MMP12 levels in BALF of C57BL/6J mice exposed to PG/VG with nicotine [33].Similarly, Wang, *et al*. determined sex-specific alterations in the expressions of MMPs and TIMP-1 in pups born from pregnant dams exposed to PG/VG with nicotine [15]. Though the changes observed in this work were contrary to our results, but it still substantiates the fact that responses towards exposure to TFN and TDN salts varies based on sex which should be an important consideration in future work. Previous work has also shown cell-specific changes in MMP production on e-cig aerosol exposure. *In vitro* exposure to flavored JUUL pods with 5% nicotine salts in lung epithelial cells (Beas2b) resulted in an upregulation of MMP12 but downregulation of MMP9 gene expression while in murine macrophages( Raw264.7) resulted in an upregulation in MMP12 and no change in MMP9 gene expression [34]. These alterations suggest the potential for lung extracellular matrix remodeling and the development of respiratory diseases caused by alterations in MMPs [35, 36].

It is pertinent to mention that there were few limitations in determining the source of nicotine used TDN salts exposure in this study. Current analysis could definitively determine the presence of R- and S-nicotine in the e-liquids prepared using TFN salts but could only determine the presence of S-nicotine in the e-liquids prepared using TDN salts. Without conducting radiocarbon analysis to determine the C^14^ content of the nicotine, the e-liquids prepared using TDN salts cannot be determined to be Tobacco-derived [5]. Furthermore, the duration of exposure for this study was too short to observe noticeable phenotypic changes within the lung. Future work with longer exposure durations might be able to shed more light on the toxicity and immune-modulators on TFN exposures in vivo. Despite these shortcomings, this study is one of the first studies to determine the effects of exposure to TFN salts in mice and also compares the differences in the inhalation of S-nicotine and TFN. Besides being one of the first *in vivo* studies on TFN, this study utilized a nose-only exposure system and a puffing profile that mimics current e-cigarette user puffing topography, allowing this study to best mimic an exposure relevant to human users.

## Conclusions

Overall, this study was able to show that exposure to the humectants, PG/VG, used in e-cigarettes alone was able to increase cytokine levels, IL-6, KC, and MCP-1 in BALF and KC levels in lung homogenate of exposed C57Bl/6J mice. It also demonstrated that despite few changes in the differential immune cell counts in the BALF and lung homogenates, the level of KC, IL6 and MCP1 remained comparable in both TFN and TDN-exposed groups. We further provide evidence of sex-specific changes in th the expression and activity of MMP9 in TFN salt exposed mouse lungs. This study also suggests that the metabolism of nicotine or the clearance of cotinine from TFN may differ from the metabolism of nicotine or the clearance of cotinine from TDN. These findings indicate that exposure of humectants alone can induce an inflammatory response while exposure to TFN or TDN salts may differentially alter inflammatory responses and lung proteases production.

## Data Availability

All data are provided in this manuscript.

## Abbreviations

ENDS: electronic nicotine delivery systems
e-cigarettes: electronic cigarette
PG: propylene glycol
VG: vegetable glycerin
TDN: tobacco-derived nicotine
TFN: synthetic nicotine
BALF: bronchoalveolar lavage fluid
MMP: matrix metalloproteinases
IL: Interleukin
KC: keratinocyte chemoattractant
MCP-1: monocyte chemoattractant protein-1
FDA: Food and Drug Administration
^1^H NMR: proton nuclear magnetic resonance
CO: carbon monoxide
TPM: total particulate matter
ELISA: enzyme-linked immunosorbent assay
FBS: fetal bovine serum
BSA: bovine serum albumin
TBST: tris-buffered saline with 0.1% tween 20
TIMP-1: tissue inhibitor of metalloproteinases-1
GAPDH: glyceraldehyde-3-phosphate dehydrogenase
mRNA: messenger ribonucleic acid
AO/PI: acridine orange/propidium iodide
LPS: lipopolysaccharide

## References

[1] Stephenson J: FDA Gains Power to Regulate Synthetic Nicotine in e-Cigarettes. JAMA Health Forum 2022, 3:e221140. 36218961 10.1001/jamahealthforum.2022.1140

[2] Zettler PJ, Hemmerich N, Berman ML: Closing the Regulatory Gap for Synthetic Nicotine Products. Boston Coll Law Rev 2018, 59:1933–1982. 30636822 PMC6329380

[3] Benowitz NL, Hukkanen J, Jacob P, 3rd: Nicotine chemistry, metabolism, kinetics and biomarkers. Handb Exp Pharmacol 2009:29–60. 19184645 10.1007/978-3-540-69248-5_2 PMC2953858

[4] Salam S, El-Hajj Moussa F, El-Hage R, El-Hellani A, Aoun Saliba N: A Systematic Review of Analytical Methods for the Separation of Nicotine Enantiomers and Evaluation of Nicotine Sources. Chem Res Toxicol 2023, 36:334–341. 36897818 10.1021/acs.chemrestox.2c00310 PMC10031562

[5] Cheetham AG, Plunkett S, Campbell P, Hilldrup J, Coffa BG, Gilliland S, 3rd, Eckard S: Analysis and differentiation of tobacco-derived and synthetic nicotine products: Addressing an urgent regulatory issue. PLoS One 2022, 17:e0267049. 35421170 10.1371/journal.pone.0267049 PMC9009602

[6] Zhang H, Pang Y, Luo Y, Li X, Chen H, Han S, Jiang X, Zhu F, Hou H, Hu Q: Enantiomeric composition of nicotine in tobacco leaf, cigarette, smokeless tobacco, and e-liquid by normal phase high-performance liquid chromatography. Chirality 2018, 30:923–931. 29722457 10.1002/chir.22866

[7] Jordt SE: Synthetic nicotine has arrived. Tob Control 2023, 32:e113–e117. 34493630 10.1136/tobaccocontrol-2021-056626 PMC8898991

[8] Gholap VV, Kosmider L, Golshahi L, Halquist MS: Nicotine forms: why and how do they matter in nicotine delivery from electronic cigarettes? Expert Opin Drug Deliv 2020, 17:1727–1736. 32842785 10.1080/17425247.2020.1814736 PMC9361466

[9] Harvanko AM, Havel CM, Jacob P, Benowitz NL: Characterization of Nicotine Salts in 23 Electronic Cigarette Refill Liquids. Nicotine Tob Res 2020, 22:1239–1243. 31821492 10.1093/ntr/ntz232 PMC7291795

[10] Shao XM, Friedman TC: Pod-mod vs. conventional e-cigarettes: nicotine chemistry, pH, and health effects. J Appl Physiol (1985) 2020, 128:1056–1058. 31854246 10.1152/japplphysiol.00717.2019 PMC7191502

[11] Keithly L, Ferris Wayne G, Cullen DM, Connolly GN: Industry research on the use and effects of levulinic acid: a case study in cigarette additives. Nicotine Tob Res 2005, 7:761–771. 16191747 10.1080/14622200500259820

[12] Adrian CL, Olin HB, Dalhoff K, Jacobsen J: In vivo human buccal permeability of nicotine. Int J Pharm 2006, 311:196–202. 16457974 10.1016/j.ijpharm.2005.12.039

[13] Mishra A, Chaturvedi P, Datta S, Sinukumar S, Joshi P, Garg A: Harmful effects of nicotine. Indian J Med Paediatr Oncol 2015, 36:24–31. 25810571 10.4103/0971-5851.151771 PMC4363846

[14] Wang Q, Khan NA, Muthumalage T, Lawyer GR, McDonough SR, Chuang TD, Gong M, Sundar IK, Rehan VK, Rahman I: Dysregulated repair and inflammatory responses by e-cigarette-derived inhaled nicotine and humectant propylene glycol in a sex-dependent manner in mouse lung. FASEB Bioadv 2019, 1:609–623. 31825014 10.1096/fba.2019-00048 PMC6902908

[15] Wang Q, Sundar IK, Blum JL, Ratner JR, Lucas JH, Chuang TD, Wang Y, Liu J, Rehan VK, Zelikoff JT, Rahman I: Prenatal Exposure to Electronic-Cigarette Aerosols Leads to Sex-Dependent Pulmonary Extracellular-Matrix Remodeling and Myogenesis in Offspring Mice. Am J Respir Cell Mol Biol 2020, 63:794–805. 32853043 10.1165/rcmb.2020-0036OC PMC7790147

[16] Muthumalage T, Lamb T, Friedman MR, Rahman I: E-cigarette flavored pods induce inflammation, epithelial barrier dysfunction, and DNA damage in lung epithelial cells and monocytes. Sci Rep 2019, 9:19035. 31836726 10.1038/s41598-019-51643-6 PMC6910911

[17] Ikushima S, Muramatsu I, Sakakibara Y, Yokotani K, Fujiwara M: The effects of d-nicotine and l-isomer on nicotinic receptors. J Pharmacol Exp Ther 1982, 222:463–470. 7097565

[18] Jacob P 3, Benowitz NL, Copeland JR, Risner ME, Cone EJ: Disposition kinetics of nicotine and cotinine enantiomers in rabbits and beagle dogs. J Pharm Sci 1988, 77:396–400. 3411460 10.1002/jps.2600770508

[19] Lamb T, Muthumalage T, Meehan-Atrash J, Rahman I: Nose-Only Exposure to Cherry- and Tobacco-Flavored E-Cigarettes Induced Lung Inflammation in Mice in a Sex-Dependent Manner. Toxics 2022, 10. 10.3390/toxics10080471 PMC9413458 36006150

[20] Duell AK, Kerber PJ, Luo W, Peyton DH: Determination of (R)-(+)- and (S)-(−)-Nicotine Chirality in Puff Bar E-Liquids by (1)H NMR Spectroscopy, Polarimetry, and Gas Chromatography-Mass Spectrometry. Chem Res Toxicol 2021, 34:1718–1720. 34196534 10.1021/acs.chemrestox.1c00192 PMC10861124

[21] Lee YO, Nonnemaker JM, Bradfield B, Hensel EC, Robinson RJ: Examining Daily Electronic Cigarette Puff Topography Among Established and Nonestablished Cigarette Smokers in their Natural Environment. Nicotine Tob Res 2018, 20:1283–1288. 29059416 10.1093/ntr/ntx222 PMC6121870

[22] Dautzenberg B, Bricard D: Real-Time Characterization of E-Cigarettes Use: The 1 Million Puffs Study. J Addict Res Ther 2015, 6:229.

[23] Moshensky A, Brand CS, Alhaddad H, Shin J, Masso-Silva JA, Advani I, Gunge D, Sharma A, Mehta S, Jahan A, : Effects of mango and mint pod-based e-cigarette aerosol inhalation on inflammatory states of the brain, lung, heart, and colon in mice. Elife 2022, 11. 10.7554/eLife.67621 PMC9005188 35411847

[24] Been T, Alakhtar B, Traboulsi H, Tsering T, Bartolomucci A, Heimbach N, Paoli S, Burnier J, Mann KK, Eidelman DH, Baglole CJ: Chronic low-level JUUL aerosol exposure causes pulmonary immunologic, transcriptomic, and proteomic changes. FASEB J 2023, 37:e22732. 36694994 10.1096/fj.202201392R

[25] Been T, Traboulsi H, Paoli S, Alakhtar B, Mann KK, Eidelman DH, Baglole CJ: Differential impact of JUUL flavors on pulmonary immune modulation and oxidative stress responses in male and female mice. Arch Toxicol 2022, 96:1783–1798. 35254488 10.1007/s00204-022-03269-3

[26] Chapman DG, Casey DT, Ather JL, Aliyeva M, Daphtary N, Lahue KG, van der Velden JL, Janssen-Heininger YMW, Irvin CG: The Effect of Flavored E-cigarettes on Murine Allergic Airways Disease. Sci Rep 2019, 9:13671. 31541174 10.1038/s41598-019-50223-y PMC6754426

[27] Ahmad S, Zafar I, Mariappan N, Husain M, Wei CC, Vetal N, Eltoum IA, Ahmad A: Acute pulmonary effects of aerosolized nicotine. Am J Physiol Lung Cell Mol Physiol 2019, 316:L94–L104. 30358437 10.1152/ajplung.00564.2017 PMC6383503

[28] Bleul T, Zhuang X, Hildebrand A, Lange C, Böhringer D, Schlunck G, Reinhard T, Lapp T: Different Innate Immune Responses in BALB/c and C57BL/6 Strains following Corneal Transplantation. J Innate Immun 2021, 13:49–59. 32906119 10.1159/000509716 PMC7879253

[29] Mabley J, Gordon S, Pacher P: Nicotine exerts an anti-inflammatory effect in a murine model of acute lung injury. Inflammation 2011, 34:231–237. 20625922 10.1007/s10753-010-9228-x PMC3008511

[30] Glynos C, Bibli SI, Katsaounou P, Pavlidou A, Magkou C, Karavana V, Topouzis S, Kalomenidis I, Zakynthinos S, Papapetropoulos A: Comparison of the effects of e-cigarette vapor with cigarette smoke on lung function and inflammation in mice. Am J Physiol Lung Cell Mol Physiol 2018, 315:L662–L672. 30091379 10.1152/ajplung.00389.2017

[31] Garcia-Arcos I, Geraghty P, Baumlin N, Campos M, Dabo AJ, Jundi B, Cummins N, Eden E, Grosche A, Salathe M, Foronjy R: Chronic electronic cigarette exposure in mice induces features of COPD in a nicotine-dependent manner. Thorax 2016, 71:1119–1129. 27558745 10.1136/thoraxjnl-2015-208039 PMC5136722

[32] Escobar YH, Nipp G, Cui T, Petters SS, Surratt JD, Jaspers I: In Vitro Toxicity and Chemical Characterization of Aerosol Derived from Electronic Cigarette Humectants Using a Newly Developed Exposure System. Chem Res Toxicol 2020, 33:1677–1688. 32223225 10.1021/acs.chemrestox.9b00490 PMC11391858

[33] Roxlau ET, Pak O, Hadzic S, Garcia-Castro CF, Gredic M, Wu CY, Schaffer J, Selvakumar B, Pichl A, Spiegelberg D, : Nicotine promotes e-cigarette vapour-induced lung inflammation and structural alterations. Eur Respir J 2023, 61. 10.1183/13993003.00951-2022 PMC10285110 37105573

[34] Pinkston R, Zaman H, Hossain E, Penn AL, Noel A: Cell-specific toxicity of short-term JUUL aerosol exposure to human bronchial epithelial cells and murine macrophages exposed at the air-liquid interface. Respir Res 2020, 21:269. 33069224 10.1186/s12931-020-01539-1 PMC7568376

[35] Culpitt SV, Rogers DF, Traves SL, Barnes PJ, Donnelly LE: Sputum matrix metalloproteases: comparison between chronic obstructive pulmonary disease and asthma. Respir Med 2005, 99:703–710. 15878486 10.1016/j.rmed.2004.10.022

[36] Gharib SA, Manicone AM, Parks WC: Matrix metalloproteinases in emphysema. Matrix Biol 2018, 73:34–51. 29406250 10.1016/j.matbio.2018.01.018 PMC6377072

